# Albumin level and progression of coronary artery lesions in Kawasaki disease: A retrospective cohort study

**DOI:** 10.3389/fped.2022.947059

**Published:** 2022-09-14

**Authors:** Yuhan Xia, Huixian Qiu, Zhengwang Wen, Hongying Shi, Huan Yu, Jie Li, Qihao Zhang, Jianjie Wang, Xing Rong, Rongzhou Wu, Maoping Chu

**Affiliations:** ^1^Children’s Heart Center, The Second Affiliated Hospital and Yuying Children’s Hospital, Institute of Cardiovascular Development and Translational Medicine, Wenzhou Medical University, Zhejiang, China; ^2^Department of Preventive Medicine, School of Public Health and Management, Wenzhou Medical University, Wenzhou, China

**Keywords:** Kawasaki disease, albumin level, coronary artery lesions progression, multiple logistic regression models, stratified analysis

## Abstract

**Background:**

Albumin (ALB) level is closely associated with the occurrence of intravenous immunoglobulin (IVIG) resistance and coronary artery lesions (CALs) in Kawasaki disease (KD). The association between ALB level and CALs progression, is critical to the prognosis of KD patients. But little is known about it. This study aims to investigate the effect of the ALB level on CALs progression in KD patients.

**Methods:**

A total of 3,479 KD patients from 1 January 2005 to 30 November 2020, in Wenzhou, China were recruited. A total of 319 KD patients who had CALs and ALB data, and finish the follow-up as requested were enrolled in this study. They were classified into the low ALB group and the normal ALB group, divided by 30 g/L. CALs outcomes were classified into two categories according to the CALs changes from the time that CALs were detected within 48 h before or after IVIG treatment to 1 month after disease onset: progressed and no progressed. Multiple logistic regression models were used to assess the independent effect of ALB level on CALs progression among KD patients. Stratified analysis was performed to verify the ALB level on CALs progression among patients in different subgroups.

**Results:**

Higher proportion of IVIG resistance (*P* < 0.001), receiving non-standard therapy (*P* < 0.001), and receiving delayed IVIG treatment (*P* = 0.020) were detected in patients with lower ALB level. Patients with lower ALB level had higher C-reactive protein (CRP) level (*P* = 0.097) and white blood cell count (WBC) (*P* = 0.036). After adjustment for confounders, patients with lower ALB level had higher odds of CALs progression; the adjusted odds ratio (OR) was 3.89 (95% CI: 1.68, 9.02). Similar results were found using stratification analysis and sensitivity analysis. Male gender and age over 36 months, as covariates in multiple logistic regression models, were also associated with CALs progression.

**Conclusion:**

Low ALB level is identified as an independent risk factor for CALs progression in KD patients. Male gender and age over 36 months are also proved to be risk factors for CALs progression. Further investments are required to explore its mechanisms.

## Background

Kawasaki disease (KD) is an acute systemic vasculitis in young children that affects medium-sized arteries, specifically the coronary arteries (CAs) (1). Coronary artery lesions (CALs) are the most common complication of KD, including coronary artery dilatations (CADs) and coronary artery aneurysms (CAAs), which are leading causes of acquired heart disease among children in developed countries (2, 3). The peak incidence of CALs is in the acute phase (4). Although treated with high-dose intravenous immunoglobulin (IVIG), about 8–16% of the patients develop CALs during the acute phase (5–9). Most of the CALs occurring in the acute phase will gradually disappear over time, but part of children with CALs will persist or progress, and lead to stenosis or obstruction, or even acute myocardial infarction (10).

A 40-year retroperspective study revealed that giant CAAs rarely regressed and were likely to cause myocardial ischemia or even sudden death, and half of the non-giant CAAs may persist, with 14–20% progressing to stenosis. The CA diameter-based severity 1 month after KD onset was found to be the most significant predictor of late coronary outcomes. However, the probable mechanism for such phenomenon remained unknown (11). Another study found that CALs progressed 1 month after the initial fever detection in 4.2% of the patients. Patients aged over 60 months and those with KD recurrent status or parental KD history might be at higher risk for worse coronary outcomes (12). However, the effect of biomarkers on CALs progression is left unexplored.

The albumin (ALB), which is traditionally regarded as a marker of nutritional status (13–15), is increasingly considered an important indicator of inflammation (16, 17). Hypoalbuminemia is commonly observed in patients with KD, which may be primarily resulted from the increased permeability and leakage of ALB during the acute phase (14, 18). Studies show that the low ALB level is related to IVIG resistance and CALs occurrence (19–21). But few studies focus on the association between ALB level and CALs progression, though this is critical to the prognosis of KD patients.

This study aims to examine whether the low ALB level before IVIG treatment is an independent risk factor affecting the CALs progression of KD patients, based on 16 years’ medical records of KD patients; and to assess the independent effect of ALB level on CALs progression after adjustment for confounders.

## Materials and methods

### Patients

**FIGURE 1:**
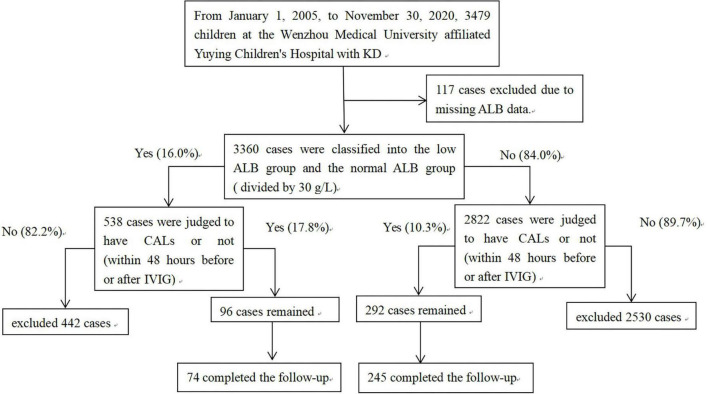
Patients flow chart. Flow chart showing the demographic and clinical information of all study participants. From 1 January 2005 to 30 November 2020, 3,479 children in our KD database were enrolled. A total of 117 cases were excluded due to missing ALB data. The remaining patients were classified into the low ALB group and the normal ALB group, divided by 30 g/L. A total of 319 patients who had CALs within 48 h before or after IVIG treatment and rechecked the echocardiography at 1 month after KD onset were included in the final analysis. KD, Kawasaki disease; CALs, coronary artery lesions; IVIG, intravenous immunoglobulin; ALB, albumin level.

Medical records for all children hospitalized with KD at the Wenzhou Medical University affiliated Yuying Children’s Hospital were reviewed. The diagnostic criteria for KD were adopted from the 2013 Japanese KD diagnostic guide (22). From 1 January 2005 to 30 November 2020, 3,479 children were enrolled. Of them, 117 patients were excluded due to lacking ALB data before IVIG treatment. The remaining 3,360 cases were divided into two groups by ALB level before IVIG treatment. A total of 538 patients with ALB level <30 g/L were regarded low ALB, the other 2,822 patients ALB level ≥30 g/L were regarded normal ALB. All patients hospitalized for KD routinely received echocardiography within 48 h before or after IVIG, for CALs screening. Patients with CALs were advised for another echocardiography at 1 month after KD onset. A total of 74 and 245 patients completed follow-up were analyzed in low ALB group and normal ALB group, respectively (Figure 1).

The complete follow-up patients were compared with lost to follow-up patients to assess selection bias. It is found that no difference in basic characteristics between them (Supplementary material 1).

This study was approved by the ethical Board of Wenzhou Medical University, Zhejiang, China. The requirement for individual consent was waived for this retrospective study.

### Assessment of coronary artery lesions

Echocardiography results were reviewed by two pediatric cardiologists independently, for screening the occurrence of CALs. Both cardiologists were blind to the patients’ medical records, hospital identification, and other clinical information. When the two cardiologists failed to reach agreement, a senior pediatric physician was requested to make a determination. CA diameter vary between different races (23). Thus, diagnostic criteria for CAD and CAA specified for Chinese children were adopted (24–27): (1) CAD: coronary artery diameter of >2.5 mm in children younger than 3 years old; >3.0 mm in children 3–9 years old; and >3.5 mm in children older than 9 years old, as well as the diameter of one segment of the coronary artery >1.5 times that of the adjacent segment; or lumen is clearly irregular. (2) Non-giant CAA was defined as lumen size of 4–8 mm or ≥1.5 times greater than that of an adjacent segment, and giant CAA as lumen size ≥8 mm. Multiple types of CALs could be diagnosed in the same patient (e.g., CAA with CAD), and these patients were categorized according to the most severe lesions (giant CAA > non-giant CAA > CAD).

**FIGURE 2:**
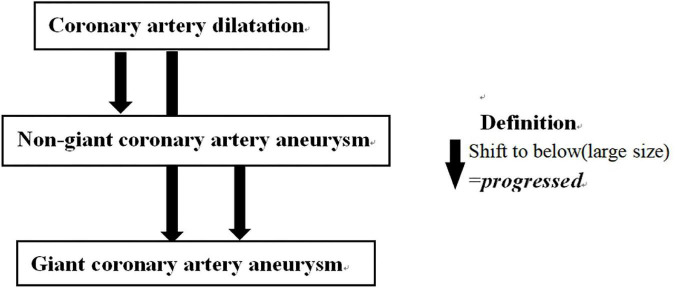
Definition of CALs outcomes. CALs outcomes were classified as CALs changes from the time that CALs were detected to 1 month after KD onset in two categories: progressed and no progressed. CALs, coronary artery lesions; KD, Kawasaki disease.

Echocardiography at 1 month after KD onset was thought not only to reflect the severity of CALs (7, 28) but also to be associated with subsequent CALs persistence and eventual ischemia events (11, 29). Thus, it was used to determine whether KD patients had CALs progression. CALs outcomes were classified into two categories according to the change of CALs from CALs were detected to 1 month after disease onset: progressed and no progressed. Patients with enlarged CAL (change from CAD to non-giant CAA or to giant CAA or from non-giant CAA to giant CAA) was defined as “progressed”, otherwise was defined as “no progressed” (Figure 2). However, due to the patient compliance, we adopted a range from 20 to 40 days after KD onset for “echocardiography follow-up at one month after onset”.

### Assessment of covariates

Kawasaki disease type: complete KD was defined as having at least four clinical manifestations in addition to persistent fever (≥5 days), and incomplete KD as having two manifestations in addition to persistent fever and CALs or having three clinical manifestations in addition to persistent fever (22).

Intravenous immunoglobulin therapeutic effect: IVIG therapeutic effect were classified as IVIG resistance and IVIG sensitive. The former was defined as persistent fever over 38.0°C 48 h after the end of IVIG infusion, otherwise is defined as the latter (30).

Treatment regimen: all patients were treated with aspirin at a dose of 30–50 mg/kg per day in acute phase. According to the total dose of immunoglobulin and weight of the children, the treatment regimen was divided into two types: the standard treatment regimen (2 g/kg, single-dose intravenous injection), and the non-standard treatment regimen (for example, 1 g/kg/day for two consecutive days).

Time of IVIG treatment: receiving IVIG treatment over 10 days after onset was defined as delayed IVIG treatment, the others was defined as non-delayed IVIG treatment.

Laboratory indices that were of a priori interest or may confounded the association of ALB level with CALs progression were included into covariates, including serum ALB, C-reactive protein (CRP), white blood cell count (WBC), platelet count (PLT), alanine aminotransferase (ALT). All laboratory indices were collected before IVIG treatment.

### Statistical analysis

The main exposure of interest was the low ALB group vs. the normal ALB group. In the secondary analysis, we also classified patients into four groups according to the level of ALB before IVIG treatment: the low ALB group (ALB was <30 g/L), the middle ALB group (ALB was 30–34.9 g/L), the high ALB group (ALB was 35–39.9 g/L) and the very high ALB group (ALB was ≥40 g/L).

First, demographics and clinical features between the low ALB patients and normal ALB patients were compared. Continuous variables were compared using two independent samples *t*-test or rank-sum test as appropriate; categorical variables were compared using the Chi-square test or Fisher test.

Multiple logistic regression models were used to assess the effect of ALB level and other factors on CALs progression. In model 1, we adjusted only age (in months) and gender. In model 2, we adjusted for age (in months), gender, KD type, IVIG therapeutic effect, treatment regimen, and time of IVIG treatment. In model 3, we additionally adjusted laboratory indices.

Stratified analysis was also performed to verify the level of ALB on CALs progression among different subgroups of patients. Moreover, sensitivity analysis for male genders and receiving non-delayed IVIG treatment patients were performed.

The statistical analyses were performed using IBM SPSS (version 25). All tests were two-tailed. *P* < 0.05 was considered statistically significant.

## Results

A total of 319 KD patients were included in this analysis. The median age was 18.6 months [interquartile range (IQR): 10.6–34.1 months], 72.4% was male, 66.5% was complete KD, 90.0% was sensitive to IVIG, 88.1% received standard IVIG regimen, and 84.3% received the non-delayed IVIG treatment.

### Demographic and clinical characteristics

Table 1 showed the basic demographics and clinical characteristics of the low ALB group patients and the normal ALB group patients. Patients with low ALB level had higher proportions of IVIG resistance (*P* < 0.001), non-standard therapy (*P* < 0.001), and delayed IVIG treatment (*P* = 0.020). Moreover, higher CRP level (*P* = 0.097) and white blood cell (WBC) count (*P* = 0.036) were observed in these patients. However, no significant differences were found in age, gender, KD type, PLT count, and ALT level.

**TABLE 1:** Basic characteristics of Kawasaki disease patients by ALB levels.

Characteristics	Low ALB group (*N* = 74)	Normal ALB group (*N* = 245)	*P*-value
Age (months)	15.2 (7.8-32.2)	19.5 (11.4-35.7)	0.127
Male, *n* (%)	55 (74.3)	176 (71.8)	0.675
Incomplete KD, *n* (%)	21 (28.4)	86 (35.1)	0.283
IVIG resistance, *n* (%)	16 (21.6)	16 (6.5)	<0.001
Non-standard IVIG treatment regimen, *n* (%)	22 (29.7)	16 (6.5)	<0.001
Delayed IVIG treatment, *n* (%)	18 (24.3)	32 (13.0)	0.020
CRP >70 mg/L, *n* (%)	50 (67.6)	139 (56.7)	0.097
WBC >18 × 109/L, *n* (%)	29 (39.2)	65 (26.5)	0.036
PLT >450 × 109/L, *n* (%)	27 (36.5)	75 (30.6)	0.342
ALT >45 U/L, *n* (%)	34 (45.9)	105 (42.9)	0.639

Quantitative data were expressed as mean ± SD and compared with the t-test if normally distributed, otherwise expressed as median (inter-quartile range) and compared with the rank-sum test, and qualitative data were expressed as frequency (%) and were compared with the Chi-square test or the Fisher exact test as appropriate.

KD, Kawasaki disease; IVIG, intravenous immunoglobulin; CRP, C-reactive protein level; WBC, white blood cell count; PLT, platelet count; ALT, alanine aminotransferase level.

### Incidence of coronary artery lesions progression

**FIGURE 3:**
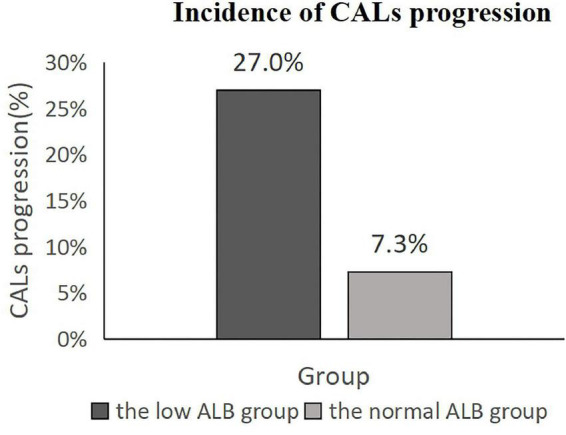
Incidence of coronary artery lesion progression among Kawasaki disease patients. The low ALB group: albumin level <30 g/L. The normal ALB group: albumin level ≥30 g/L. CALs, coronary artery lesions; ALB, albumin level.

The total incidence of CALs progression was 11.9% (38/319). In total, 20 cases of which were from the low ALB group (17 cases progressed from CAD to non-giant CAA and 3 cases progressed from non-giant CAA to giant CAA), and the other 18 cases had normal ALB level (17 cases progressed from CAD to non-giant CAA and 1 case progressed from non-giant CAA to giant CAA). The incidence of CALs progression in the low ALB group and the normal ALB group were 27.0% (20/74) and 7.3% (18/245), respectively (*P* < 0.001) (Figure 3).

### The independent effect of albumin level on the coronary artery lesions progression

Table 2 showed the adjusted odds ratios (ORs) of CALs progression by the ALB status. Initially, we used the ALB status (two groups) as the independent variable. In model 1 we adjusted only age and gender, and found that compared with the normal ALB group, the low ALB group was associated with increased odds of CALs progression (adjusted OR = 5.19, 95% CI: 2.48, 10.87). In model 2, we additionally adjusted KD type, IVIG therapeutic effect, treatment regimen, and time of IVIG treatment into the covariates, low ALB level was still associated with increased odds of CALs progression (adjusted OR: 4.43, 95% CI: 1.95, 10.06). In model 3, we found that additional adjustment for laboratory indices only minimally changed the observed association (adjusted OR: 3.89, 95% CI: 1.68, 9.02). In secondary analysis, we divided the normal ALB group into three subgroups and compared with the low ALB group respectively (middle ALB, high ALB, and very high ALB level for 30–34.9, 35–40, and over 40 g/L, respectively). It was found that the odds of CALs progression were much larger when compared the low ALB group with the very high ALB group, which remind a dose-response relationship between ALB level and CALs progression. In model 1, for instance, in comparison with patients with low ALB level, the adjusted ORs for CALs progression among the patients with middle, high, and very high ALB level were 4.46 (95% CI: 1.37, 14.52), 3.95 (95% CI: 1.63, 9.57) and 9.56 (95% CI: 3.02, 30.29) respectively. Similar conclusions were observed when using model 2 and model 3 (Table 2).

**TABLE 2:** Independent effect of ALB level on CALs progression among Kawasaki disease patients.

Exposure	OR (95% CI) *P*-value		
	Model 1	Model 2	Model 3
**ALB level (2 groups)**			
≥30 g/L (*n* = 245)	1 (reference)	1 (reference)	1 (reference)
<30 g/L (*n* = 74)	5.19 (2.48, 10.87) <0.001	4.43 (1.95, 10.06) <0.001	3.89 (1.68, 9.02) 0.002
**ALB level (4 groups)**			
30–34.9 g/L (*n* = 56)	1 (reference)	1 (reference)	1 (reference)
<30 g/L (*n* = 74)	4.46 (1.37, 14.52) 0.013	4.02 (1.12, 14.45) 0.033	4.46 (1.16, 17.10) 0.029
35–40 g/L (*n* = 103)	1 (reference)	1 (reference)	1 (reference)
<30 g/L (*n* = 74)	3.95 (1.63, 9.57) 0.002	3.54 (1.35, 9.29) 0.010	3.24 (1.20, 8.73) 0.020
≥40 g/L (*n* = 86)	1 (reference)	1 (reference)	1 (reference)
<30 g/L (*n* = 74)	9.56 (3.02, 30.29) <0.001	8.60 (2.44, 30.28) 0.001	5.88 (1.56, 22.18) 0.009

ALB, serum albumin; IVIG, intravenous immunoglobulin; CALs, coronary artery lesions; OR, odds ratio.

Model 1 adjusted for: age (months, ≤36 months, >36 months), and gender (male, female).

Model 2 adjusted for: model 1+ Kawasaki disease type (complete, incomplete), IVIG therapeutic effect (sensitive, resistance), treatment regimen (standard, non-standard), and time of IVIG treatment (delayed, non-delayed).

Model 3 adjusted for: model 2+ C-reactive protein level (≤70 mg/L, >70 mg/L), white blood cell count (≤18 × 109/L, >18 × 109/L), platelet count (≤450 × 109/L, >450 × 109/L), and alanine aminotransferase level (≤45 U/L, >45 U/L).

The logistic regression revealed that OR for CALs progression increased with proportion of male gender and patients age over 36 months. The adjusted ORs for CALs progression with male gender were 7.56 (95% CI: 1.74, 32.78),6.85 (95% CI: 1.57, 29.92), and 6.57 (95% CI: 1.48, 29.21) in model 1, model 2, and model 3, respectively. The adjusted ORs with age over 36 months were 2.20 (95% CI: 1.01, 4.81), 2.28 (95% CI: 1.03, 5.03) and 2.52 (95% CI: 1.08, 5.90) in model 1, model 2 and model 3, respectively.

### Stratified analysis and sensitivity analysis

Stratified analysis showed that the patients with low ALB level had higher risk for CALs progression. Although the adjusted ORs were not statistically significant in several subgroups because of the small sample size (Table 3). Separate analysis for the female gender, delayed IVIG treatment patients, non-standard treatment regimen patients, resistant to IVIG treatment patients, and incomplete KD patients was not performed because of the small number. Therefore, we restricted the participants within male patients, non-delayed IVIG treatment patients, standard treatment regimen patients, sensitive to IVIG treatment patients, and complete KD patients separately; adjusted ORs were 4.45 (95% CI: 1.83, 10.80), 3.05 (95% CI: 1.24, 7.53), 3.95 (95% CI: 1.59, 9.82), 3.75 (95% CI: 1.54, 9.10), and 2.65 (95% CI: 1.00, 7.06) respectively.

**TABLE 3:** Stratified analysis of the effects of ALB levels on CALs progression among Kawasaki disease patients.

Stratification factor	*N*	All patients (n = 319)	
		OR (95% CI)*	*P*-value
Age (months)			
≤36	244	4.32 (1.49, 12.54)	0.007
>36	75	3.89 (0.79, 19.02)	0.094
CRP (mg/L)			
≤70	130	14.42 (0.89, 234.87)	0.061
>70	189	2.44 (0.95, 6.30)	0.065
WBC (×109/L)			
≤18	225	1.92 (0.61, 6.018)	0.264
>18	94	11.44 (2.29, 57.10)	0.003
PLT (×109/L)			
≤450	217	1.93 (0.63, 5.87)	0.249
>450	102	10.41 (2.26, 48.03)	0.003
ALT (U/L)			
≤45	180	3.22 (0.96, 10.79)	0.058
>45	139	5.68 (1.44, 22.30)	0.013

CALs, coronary artery lesions; KD, Kawasaki disease; CRP, C-reactive protein; WBC, white blood cell; PLT, platelet count; ALT, alanine aminotransferase.

*Adjusted for: age (months), gender (male, female), KD type (complete, incomplete), IVIG therapeutic effect (sensitive, resistance), treatment regimen (standard, non-standard), time of IVIG treatment (delayed, non-delayed), C-reactive protein level (≤70 mg/L, >70 mg/L), white blood cell count, platelet count (≤450 × 109/L, >450 × 109/L), and alanine aminotransferase level (≤45 U/L, >45 U/L).

## Discussion

Patients with low ALB level are found to have a higher risk for CALs progression during the acute phase. However, some risk factors for CALs progression clusters in the low ALB level patients, and it will increase the probability of CALs progression. We take it into consideration that it due to the physiological or pathological mechanisms of patients with low ALB level.

First of all, ALB level is an effective biomarker for measuring the activity of inflammatory conditions (31, 32). Patients experiencing more severe inflammatory response during the acute phase have higher vascular permeability, resulting in lower ALB level. Such severe inflammatory response may also result in higher CRP level and WBC count. It is widely accepted that the formation of CALs and CAAs in KD is closely related to inflammation (10, 33). CRP level is found to have a positive association with the size of CALs and is an independent factor affecting the persistence of CALs (34, 35); and high WBC count is associated with cardiac sequelae in KD patients (36). Severe inflammation leads to further aggravation of CA damage in the acute stage and subsequent CALs progression. However, after adjusting with CRP and WBC, the low ALB level also proved to be a significant risk factor for CALs progression.

Besides, ALB level is found to be associated with IVIG resistance and is included in several risk-scoring systems for IVIG resistance prediction in KD (18, 37–40). Patients with low ALB level are also found to have a higher proportion of IVIG resistance in our study. IVIG resistance is proved that is a characteristic of severe cases and is related to the occurrence of CALs in substantial evidence (41–44). During the follow-up of KD patients with CALs, it is found that IVIG resistance is not only an independent factor for the worse echocardiography outcomes 1 month after KD onset (12), but also an independent risk factor for the persistence of CALs (45). It is also part of the reason why the proportion of CALs progression in the low ALB group is significantly higher than that in the normal ALB group. IVIG therapeutic effect is adjusted in analysis to reduce the impact on results from the association between IVIG resistance and low ALB level. The results were found change very small after adjusting it.

In addition, the proportion of delayed IVIG treatment is higher in the low ALB group, which can be explained with continued leakage of ALB before treatment. Delayed IVIG treatment is found to increase the risk of CALs (46, 47). Such relationship may amplify the effect of low ALB level on CALs progression among KD patients. A total of 2 g/kg single dose of IVIG is now the gold standard treatment in KD, which significantly reduce the risk of CAAs (3, 48). Patients with low ALB level have a higher proportion of non-standard therapy at baseline, which may lead to the increased probability of CALs progression. However, the conclusion about the effect of the low ALB level on CALs progression is still stable after controlling the time of IVIG treatment and treatment regimen.

Others factors were also found to be closely related to CALs progression. Extremes of the age spectrum was a risk factor for CA abnormalities (49–52). Patients aged no less than 60 months were thought to be at higher risk for worse coronary outcomes (14). While, age over 36 months were found to be a risk factor for CALs progression in our study. Male gender was found to be related to higher risk for KD and CALs (3). Our previous study found that the male gender was an independent risk factor for longer-lasting CALs (24). CALs in male patients were more likely to progress, according to this study.

However, after adjustment for these all factors, patients with the low ALB level are found to have higher risks for CALs progression. This proved that the low ALB level is an independent risk factor for CALs progression.

Patients with giant CAAs have a significantly higher risk of cardiovascular complications (52). Myocardial ischemia or the development of critical stenosis can cause sudden death (3). Patients with low ALB level should receive more frequent echocardiography in acute period to avoid cardiovascular complications caused by untimely detection or intervention of non-giant CAAs and giant CAAs. In terms of treatment, patients at higher risk for CAAs may benefit from adjunctive therapies for primary treatment such as corticosteroids, infliximab, plasma exchange, and cytotoxic agents (3, 41, 49). KD patients with low ALB level are believed to experience more severe inflammation, have a higher probability of IVIG resistance, and have a higher incidence of CALs progression. Thus, earlier adjunctive therapies for primary treatment may improve their outcomes.

This study has certain limitations. Firstly, there may be a delay in CALs detection during routine follow-up, due to the lack of daily echocardiography. Secondly, *Z*-score was not routinely calculated in our hospital until 2015, thus further statistical analysis is limited. Thirdly, this is a single-centered study, it is unclear whether our conclusion could be extrapolated to other population.

## Conclusion

In summary, low ALB level is identified as an independent risk factor for CALs progression in KD patients. This association is consistent in different subgroups. Male gender and age over 36 months are also proved to be risk factors for CALs progression. Further investments are required to explore the mechanisms.

## Data availability statement

The raw data supporting the conclusions of this article will be made available by the authors, without undue reservation.

## Ethics statement

The studies involving human participants were reviewed and approved by local ethics committee of the Second Affiliated Hospital and Yuying Children’s Hospital, China. Written informed consent from the participants’ legal guardian/next of kin was not required to participate in this study in accordance with the national legislation and the institutional requirements.

## Author contributions

YX and HQ were involved in conceptualization, formal analysis, investigation, methodology, validation, visualization, and writing – original draft, review, and editing. ZW was involved in writing – review and editing. HS was involved in data curation, methodology, and writing – original draft, review, and editing. HY was involved in methodology and writing – review and editing. JL, QZ, and JW were involved in data curation. XR was involved in project administration. RW was involved in resources and supervision. MC was involved in conceptualization, funding acquisition, resources, supervision, and writing – review and editing. All authors read and approved the final manuscript.
