# Supplementary material for: Albumin level and progression of coronary artery lesions in Kawasaki disease: A retrospective cohort study
Source: Front Pediatr. 2022 Sep 14;10:947059. doi: 10.3389/fped.2022.947059 (PMC9516112; doi:10.3389/fped.2022.947059)
Supplement: Supplementary file 1 [file Table_1.DOCX]

Supplementary Material 1

Basic characteristics of follow-up patients and missing patients

| Characteristics | follow-up group (N=319) | missing group (N=69) | *P value* |
| --- | --- | --- | --- |
| ALB (g/L) | 36.5 (30.3-40.2) | 35.8 (28.5-39.6) | 0.258 |
| Age (months) | 18.6(10.6-34.1) | 21.3(10.7-28.9) | 0.946 |
| CRP (mg/L) | 78.6(45.9-115.0） | 68.0(42.7-140.5) | 0.894 |
| WBC (18×109/L) | 15.7±6.0 | 16.8±6.3 | 0.172 |
| PLT (×109/L) | 369(286-490) | 365(291-520) | 0.617 |
| ALT (U/L) | 36(19-88) | 35(19-96) | 0.786 |
| Male, n (%) | 231(72.4) | 52(73.2) | 0.617 |
| Complete KD, n (%) | 212(66.4) | 45(63.4) | 0.843 |
| IVIG sensitive, n (%) | 287(90.0) | 62(87.3) | 0.977 |
| Standard IVIG treatment  regimen, n (%) | 281(88.1) | 56(78.9) | 0.122 |
| Non-delayed IVIG treatment, n (%) | 269(84.3) | 67(94.4) | 0.005 |

Quantitative data were expressed as mean ± SD and compared with the t-test if normally distributed, otherwise expressed as median (inter-quartile range) and compared with the rank-sum test, and qualitative data were expressed as frequency (%) and were compared with the Chi-square test or the Fisher exact test as appropriate.

ALB: albumin level; IVIG: Intravenous immunoglobulin; CRP: C-reactive protein level; WBC: white blood cell count; PLT: platelet count; ALT: alanine aminotransferase level.
